# A Quality Improvement Campaign Reduces Head and Cervical Spine Imaging in Low-risk Trauma Patients

**DOI:** 10.5811/westjem.50707

**Published:** 2026-07-16

**Authors:** Jude C. Luke, Rebecca N. Kubick, Heidi J. Sucharew, Kayla Winkler, Mary L. Giles, Natalie E. Kreitzer, Anita L. Goel, David O. Thompson

**Affiliations:** * University of Cincinnati College of Medicine, Cincinnati, Ohio; † University of Cincinnati, Department of Emergency Medicine, Cincinnati, Ohio; ‡ University of Cincinnati Health, Office of Performance Improvement, Cincinnati, Ohio

## Abstract

**Introduction:**

While computed tomography (CT) is invaluable for detecting life-threatening injuries in the emergency department (ED), the literature shows increasing use of head CT and cervical spine CT in low-risk trauma patients without a corresponding rise in injuries identified. These low-yield exams expose patients to avoidable radiation, increase costs, and contribute to inefficiencies in already overburdened EDs. In this study we evaluated the impact of a multimodal quality improvement (QI) campaign to reduce the use of head and cervical spine CT in low-risk trauma patients.

**Methods:**

This prospective QI intervention involved two EDs within one healthcare network: an academic trauma center and a community hospital. We collected data on baseline CT use for six months, followed by a 23-month intervention period. Primary outcomes were monthly rates of low-risk trauma head and cervical spine CT per 100 ED patients; secondary outcome was the rate of all-cause CT use per 100 ED patients. We plotted rates over time using u-charts with mean-shift lines. Pre- and post-shift differences were summarized as rate ratios (RR) with 95% confidence intervals, compared using the Poisson test.

**Results:**

Use of head CT for low-risk trauma decreased from 5.00 to 4.01 per 100 patients at the academic site (RR = 0.80, 95% CI, 0.75–0.86, *P* < .001) and from 6.64 to 5.12 per 100 patients at the community site (RR 0.77, 95% CI, 0.73–0.82, *P* < .001). Use of cervical spine CT for low-risk trauma decreased from 3.36 to 2.83 per 100 patients at the academic site (RR = 0.84, 95% CI, 0.78–0.91, *P* < .001) and from 4.31 to 3.49 at the community site (RR = 0.81, 95% CI, 0.75–0.87, *P* < .001). All-cause CT use decreased at the academic site from 53.06 to 47.91 CT exams per 100 patients [RR = 0.90, 95% CI, 0.89–0.92, *P* < .001] but remained unchanged at the community site.

**Conclusion:**

A structured quality improvement campaign led to a significant reduction in the use of head and cervical spine CT in low-risk trauma patients. These findings provide a framework for promoting evidence-based imaging practice.

## INTRODUCTION

United States emergency departments (EDs) see approximately 2.5 million head injuries annually, many of which are mild traumatic brain injuries.^1^ Computed tomography (CT) imaging is highly effective in detecting acute intracranial injury and is the recommended tool for evaluation of traumatic head and cervical spine injuries.^2^ Several studies have reported on the substantial increase in the use of CT in EDs in recent decades, including increasing use to evaluate head and cervical spine injuries.^3^–^9^ The National Hospital Ambulatory Medical Care Survey found that from 1995–2007 the number of U.S. ED visits that included a CT exam increased from 2.7 million to 16.2 million—a 5.9-fold increase—without a reciprocal increase in the prevalence of life-threatening conditions or hospital admissions.^3^,^10^ This suggested overuse of CT exposes patients to increased cumulative ionizing radiation and imposes excess financial and logistic burdens on the healthcare system.^11^–^13^

Recognizing this, the American College of Emergency Physicians identified reduction in the use of head CT in low-risk trauma patients as one of the top initiatives in their 2015 *Choosing Wisely* campaign.^14^ The problem of CT overuse, both all-cause and for low-risk trauma patients, has nevertheless persisted. A 2021 study of 305 EDs found that up to 72% of patients with minor head injuries were receiving head CTs, and suggested quality improvement (QI) initiatives to improve imaging stewardship and reduce low-value care.^4^ Another study found that over a third of head CT exams performed for trauma in the ED are likely not indicated.^15^ A separate study conducted at a Level I trauma center reported that 16% of cervical spine CT exams ordered did not have an indication according to clinical decision rule (CDR) criteria. Indeed, among these seemingly unnecessary cervical spine CT exams, no cervical injuries were identified.^16^

Several validated CDRs exist to reduce head and cervical spine imaging in low-risk trauma patients. These include the Canadian CT Head Rules (CCHR), New Orleans Criteria, and NEXUS (National Emergency X-Radiography Utilization Study) Criteria for head injuries, as well as the Canadian C-Spine Rules and NEXUS Criteria for C-spine injuries. Despite these CDRs being widely available and easy to apply, significant heterogeneity continues to be observed in the rates of head and cervical spine CT exams ordered by individual emergency physicians.^17^–^21^ This presents an opportunity to bridge the gap between evidence-based guidelines and current clinical practice patterns by investigating the factors behind these discrepancies and implementing strategies to resolve these clinical variations.^22^–^23^

Existing literature on successful ED initiatives to reduce the use of head CT^24^–^28^ and cervical spine CT^29^–^30^ exams in low-risk adult trauma patients has emphasized the paramount importance of facilitating access to and understanding of CDRs. One multicenter simulation-based randomized controlled trial identified that engaging clinicians in evidence-based education on three topics—CDRs, clinician fears of malpractice litigation, and the institutional and patient costs of excess CT use—was sufficient to precipitate a 21.1% reduction in CT ordering.^31^ The importance of patient education has also been emphasized, given that a common motivator behind unnecessary CT ordering is patient insistence.^24^ Across both the adult and pediatric interventions reviewed, the most consistent contributors to successful reductions in the use of head and cervical spine CT in low-risk trauma patients are computer-assisted clinical decision support, clinician and patient education, and performance feedback.^24^–^40^ Emergency clinician surveys and reports of unsuccessful attempts to reduce head and cervical spine CT imaging for low-risk trauma patients have identified patient expectations, clinician confidence, medicolegal concerns, and ineffective education efforts as influential barriers to compliance with established CDRs in practice.^41^, ^42^

**Population Health Research Capsule**

What do we already know about this issue?

*Overuse of head and cervical computed tomography (CT) in low-risk trauma patients exposes patients to avoidable radiation while increasing costs without benefit*.

What was the research question?

*Can a quality improvement (QI) campaign safely reduce head and cervical CT use in low-risk trauma patients?*

What was the major finding of the study?

*Per 100 patients, head CT use decreased from 5.00 to 4.01 at the academic ED, and from 6.64 to 5.12 at the community ED, while cervical CT decreased from 3.36 to 2.83 (academic) and 4.31 to 3.49 (community), all p < .001*.

How does this improve population health?

*A QI campaign led to a significant reduction in head and cervical CT exams in low-risk trauma patients, promoting safety and cost effectiveness*.

Prior to initiating this study, routine attending physician reviews within our health system revealed wide inter-clinician variability in rates of all-cause CT per 100 patients, with head CT, cervical spine CT, and CT abdomen/pelvis representing the most frequently ordered studies. Because validated CDRs exist to guide appropriate head and cervical spine CT use in trauma cases, we hypothesized that these two high-volume modalities would be the most amenable to standardized initiatives aimed at reducing practice variability. Therefore, in this study we focused on low-risk trauma patients, with the purpose of evaluating a multimodal QI initiative in reducing the use of head and cervical spine CT in these patients. We sought to achieve this goal by targeting driving factors behind our variable clinician practice patterns, especially in circumstances when CT was ordered outside CDR indications.

## METHODS

### Study Design and Rationale

We conducted a prospective, dual-site QI campaign designed to reduce the use of head and cervical spine CT among low-risk adult trauma patients in the ED. For this study we employed a pre/post intervention model that incorporated baseline assessment of CT-ordering patterns, survey-based identification of barriers to adherence with evidence-based CDRs, and implementation of targeted interventions to address these barriers. Baseline clinician CT-ordering patterns were obtained retrospectively over the 6-month pre-intervention period; survey results and subsequent ordering data were collected prospectively during the intervention period.

### Study Setting and Clinician Population

This study was conducted simultaneously across two EDs within a Midwest U.S. healthcare network. The first site is an urban academic tertiary ED and Level I trauma center with approximately 60,000 patient encounters annually. The second site is a community hospital ED and Level III trauma center with approximately 40,000 patient encounters annually. Clinicians at both EDs received information about the study followed by consent to participate. Consenting clinicians included 66 board-certified emergency medicine faculty physicians, 21 advanced practice clinicians (APC), and 63 resident/fellow physicians training in emergency medicine. Patients in the study were either seen independently by an attending physician or jointly by an attending with a resident physician or APC. The 23-month continuous intervention period lasted from July 2022–June 2024. Institutional review board approval was obtained prior to the start of the study.

### Patient Inclusion Criteria

**Figure 1:**
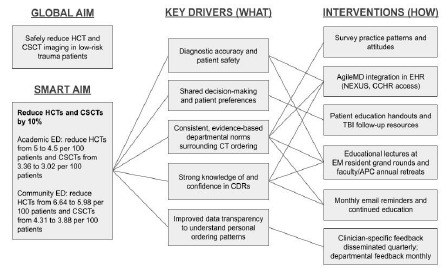
This diagram of performance improvement methodology summarizes the specific goals of the quality improvement campaign, the drivers of computed tomography use, and interventions proposed to reduce use. *APC*, advanced practice clinician*; CCHR*, Canadian CT Head Rule, CDR, clinical decision rule; *CSCT*, cervical spine computed tomography; *EHR*, electronic health record; *HCT*, head computed tomography; *NEXUS*, National Emergency X-ray Utilization Study; *TBI*, traumatic brain injury.

**Figure 2:**
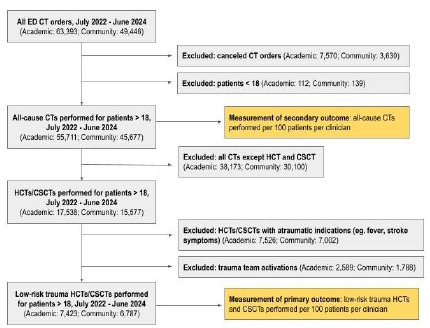
Inclusion and exclusion criteria for primary and secondary analyses in a study evaluating the impact of a quality improvement campaign to reduce use of head and cervical spine computed tomography in low-risk trauma patients. *CSCT*, cervical spine computed tomography: *CT*, computed tomography; *HCT*, head computed tomography.

Inclusion and exclusion criteria are summarized in Figure 2. Coinciding with the intervention period, all patients ≥ 18 years of age who received any type of CT in either ED between July 2022–June 2024 were considered for inclusion in the study. We collected data from the electronic health record, excluding patients < 18 years of age and patients whose CT orders were canceled. As noted in Figure 1, this initial group of patients and CT orders made up the secondary outcome of all-cause CT per 100 ED patients seen.

We identified the primary analysis cohort of low-risk trauma patients from this list of adult patients with all-cause CT, using the following exclusion criteria: patients without a head or cervical spine CT; and patients who were determined to not be low-risk trauma. Low-risk trauma patients were defined using chief complaint and primary diagnosis. Patients with “stroke symptoms” or “fever,” for example, were categorized as atraumatic, while patients with “MVC” (motor vehicle collision) or “stab wound” were categorized as traumatic. We excluded atraumatic cases from the primary analysis cohort. Patients with any trauma surgery activation were also excluded because these cases are defined by high-risk trauma features. The remaining patients were classified as low risk for trauma and included in the primary analysis.

### Pre-Intervention Survey Administration

To identify drivers of CT ordering, we distributed two brief cross-sectional surveys to all 150 clinicians in both EDs; one survey focused on head CT practice patterns and the other on cervical spine CT practice patterns. Surveys were administered via REDCap data capture software and included multiple-choice and open-response items assessing use of CDRs, typical imaging approaches, and perceived system-level barriers. Response rates were 52% for the head CT survey and 39% for the cervical spine CT survey. Responses were reviewed by blinded investigators, and we used qualitative themes to inform selection of QI interventions and shape the educational content delivered during the campaign.

### Interventions

The QI team organizing this campaign consisted of two emergency physicians, two performance improvement specialists employed by the health system, and a data analyst. Based on themes derived from clinician survey results, key drivers of unnecessary CT ordering were identified, and interventions were proposed to target each driver. For example, clinicians often expressed feeling pressured to order studies based on patient preference. To address this, the QI team created informative handouts to facilitate evidence-based discussions with patients regarding CT indications. Each key driver identified through the clinician survey was addressed similarly. The team worked together to choose interventions that were easy to implement administratively, straightforward to track, and would not be cumbersome to deploy among a large group of clinicians. The key drivers identified and corresponding interventions are outlined in Figure 1.

In addition to survey results, the QI campaign was informed by literature on similar successful campaigns. To encourage clinician adherence to validated CDRs, we embedded clinical decision support software (AgileMD Inc, San Francisco, CA) within the EHR, rather than hard-stop best practice advisories (BPAs). Education on the use of AgileMD was delivered via email and departmental presentations.

This QI campaign had a strong clinician education focus to standardize knowledge and care patterns. Clinician education efforts consisted of presentations on elements of CDRs, evidence behind the creation of these rules, baseline CT rates, and explanations of the interventions proposed to decrease the use of CT for low-risk trauma. To ensure that the educational content reached all clinicians, these presentations were given at the annual faculty retreat (approximately 80% in attendance), the annual APC retreat (70% in attendance), and during emergency medicine grand rounds, which are well attended by residents and faculty. Additionally, continued education content and reminders about the goals of the campaign were sent to clinicians by email monthly. Throughout the intervention period, individualized CT-ordering data was sent to clinicians quarterly, and departmental data were shared monthly. The CT-ordering data were always de-identified prior to dissemination to protect individual privacy. Individualized reports allowed each clinician to see where they stood in relation to their peers, with peers anonymized.

### Safety Review

To assess the safety of our interventions, investigators identified all patients (including nontrauma) over the course of the study who had 72-hour return ED visits resulting in admission; the focus was on missed injuries of the head or cervical spine on the first visit. They manually reviewed charts from the second visit to determine whether head or cervical spine injuries were identified on imaging that had not been performed during the initial visit. Patient encounters with a head or cervical spine CT obtained on their second visit underwent manual chart review to determine whether a head or cervical spine injury was discovered on imaging that had not been not discovered on the first visit due to lack of CT ordering. We tallied the total raw number of patients with a missed head or cervical spine injury.

### Costs

We calculated the cost of the QI campaign by estimating the hours spent by the faculty leads, the data analyst, and the QI analysts along with an average salary rate for each. The total annual cost was calculated as the sum of the hours spent multiplied by estimated hourly pay. Cost also included the annual software license fee multiplied by percentage attributable to this project. We estimated the average reduction in total patient charges by multiplying the cost of a CT by the reduction in CT orders observed.

### Statistical Analysis

We stratified analyses by academic and community ED sites. Demographics were summarized using count (percentage) for age, sex, race, and payor groups. The primary outcomes were monthly rates of low-risk trauma head CT and cervical spine CT per 100 patient encounters in the ED. The secondary outcome was the all-cause CT rate per 100 ED patient encounters.

For the primary outcome, outcome measures were displayed as u-charts, appropriate for count data. Interventions were noted throughout the u-charts by date of initiation so that any subsequent changes in CT ordering practices could be visualized. The centerline was plotted as the mean of the measure with baseline as the first 10 months (January–October 2022). The lower and upper control limits were estimated at three standard deviations below or above the centerline using a Poisson distribution. Based on standard QI methodology, a shift in the mean was indicated when there were eight points in a row on the same side of the center line. At these points, the center line and control limits were recalculated. For outcome measures with an observed shift in the mean, we compared the baseline rate to the shifted rate using a Poisson test along with the calculated rate ratio (RR) and 95% confidence interval. A similar analysis was performed for the secondary analysis of all-cause CTs ordered at academic and community sites. All analyses were performed using Excel v2408 (Microsoft Corp, Redmond, WA) with QI macro and SAS v9.4 (SAS Institute Inc, Cary, NC).

Demographic data of patients in both departments was determined retrospectively, including data that preceded the interventions to capture baseline characteristics. Demographic analysis extended beyond the intervention period to capture data that could be timely and relevant to conclusions.

## RESULTS

Among low-risk trauma patients with head CT, the percentage of those > 65 years of age was higher in the community ED (70.1%) than the academic site (33.3%) and for cervical spine CT the percentage of patients > 65 years of age was 23.2% community and 19.4% academic. Relative percent declines in low-risk trauma HCTs were 19.8% and 15.77% at the academic and community EDs, respectively.

**Figure 3:**
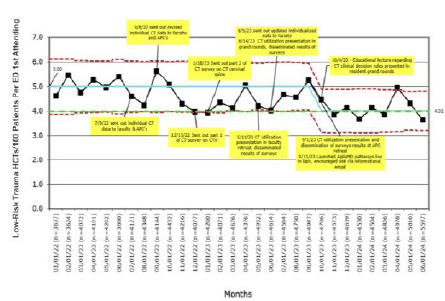
Rate of computed tomography for head trauma per 100 patient encounters, by month, in an academic ED. Quality improvement campaign intervention milestones are noted temporally along the chart. *ED*, emergency department*; HCT*, head computed tomography.

**Figure 4:**
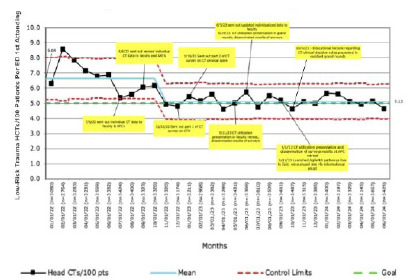
Rate of computed tomography for head trauma per 100 patient encounters in a community ED by month. Quality improvement campaign intervention milestones are noted temporally along the chart. Relative percentage declines in low-risk trauma head CTs were 19.8% and 15.77% at the academic and community EDs, respectively. *APC*, advanced practice clinician*; ED*, emergency department*; HCT*, head computed tomography.

Over the 23-month intervention period, there was a statistically significant reduction in low-risk trauma head CT and cervical spine CT ordering at both our academic and community ED sites. The mean rate of low-risk trauma head CT per 100 patients was reduced from 5.00 to 4.01 at the academic site (RR = 0.80, 95% CI, 0.75–0.86, *P* < .001) and from 6.64 to 5.12 at the community site (RR = 0.77, 95% CI, 0.73–0.82, *P* < .001) (Figures 3 and 4).

**Figure 5:**
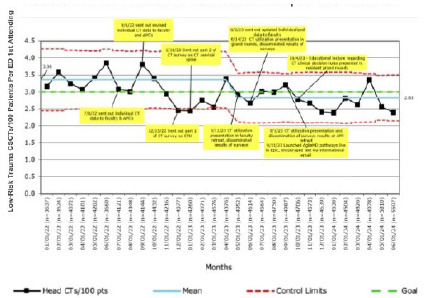
Rate of trauma cervical computed tomography per 100 patient encounters at the academic ED by month. Quality improvement campaign intervention milestones are noted temporally along the chart. *CT*, computed tomography; *ED*, emergency department.

**Figure 6:**
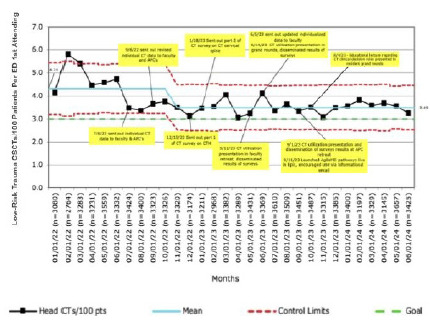
Rate of trauma cervical spine computed tomography exams per 100 patient encounters at a community ED by month. Quality improvement campaign intervention milestones are noted temporally along the chart. *APC, advanced practice clinician; CT*, computed tomography; *ED*, emergency department.

The mean rate of low-risk trauma cervical spine CT per 100 patients was reduced from 3.36 to 2.83 at the academic site (RR = 0.84, 95% CI, 0.78–0.91, *P* < .001) and from 4.31 to 3.49 at the community site (RR = 0.81, 95% CI, 0.75–0.87, *P* < .001) (Figures 5 and 6). Low-risk trauma cervical spine CTs were reduced by 15.77% at the academic ED and 19.02% at the community ED.

At the academic site, the intervention resulted in an estimated 594 fewer low-risk trauma head CT exams and 318 fewer low-risk trauma cervical spine CT exams per year. At the community site, the intervention resulted in an estimated 608 fewer low-risk trauma head CT exams and 328 fewer low-risk trauma cervical spine CT exams per year. For the secondary analysis, there was a statistically significant reduction in all-cause CT use at the academic site from 53.06 to 47.91 per 100 patients (RR = 0.90, 95% CI, 0.89–0.92, *P* < .001), corresponding with a relative reduction of 9.71%. All-cause CT use at the community site remained unchanged from the baseline value of 49.6 per 100 patients. As per our safety review of all patients who returned to the ED after a traumatic injury within 72 hours and subsequently required admission, we identified no dangerous missed head or cervical spine injuries during our study.

The estimated cost of the QI campaign was $20,000 annually. Two academic ED faculty spent 5–10 hours per month on the project. The data analyst spent 1–2 days per month extracting and organizing the CT data. The QI specialists spent 1–3 hours per month on the project. Software licensing to embed clinical pathways of CDRs into the EHR also carried associated costs, which are included in this estimate. However, the campaign resulted in 1,848 fewer CTs ordered annually. At an average cost of $1750 per CT, this means we avoided approximately $3,234,000 per year in unnecessary patient charges through our investment.

## DISCUSSION

Our results demonstrated a statistically significant reduction in low-risk trauma head CT and cervical spine CT ordering at both our academic and community EDs. We additionally found that all-cause CT use remained stable over the study period at the community site and even decreased slightly at the academic site. Given that the natural history of CT use has been a general increase over time based on reports in the literature,^3^–^10^ our data suggest that our QI campaign was a driving factor behind these observed trends in the secondary outcome of all-cause CT reduction. Higher rates of low-risk trauma head and cervical spine CT observed at our community site compared to the tertiary Level I trauma center could in part be due to an older patient population; however, this study was not designed to compare academic and community CT rates. Further research would be needed to explain these findings more conclusively.

Review of the u-charts and timing of mean shifts do not point to any one single intervention that we can attribute to a reduction in CT use; rather, the effect was likely cumulative. At the academic site, shifts in low-risk trauma patient head and cervical spine CT rates occurred after we began providing more frequent individual feedback and delivering educational lectures at faculty, APC, and resident meetings. In contrast, at the community site, shifts in the means occurred a year earlier. Because this site is primarily staffed by attending physicians and senior residents, the earlier change may reflect a Hawthorne effect following departmental announcements that low-risk trauma CT reduction would soon be a priority. However, this effect alone would likely produce only a temporary shift. The sustained change observed at both sites is more consistent with the combined impact of our QI interventions, including education, regular clinician feedback, and embedded clinical decision support. These components align with strategies used in other successful adult and pediatric QI campaigns.^40^

In comparison to the existing literature on the topic, our approach to implementation of CDRs in the EHR differed from prior studies on CT reduction. Specifically, many previous QI efforts in both the pediatric and adult literature rely on “hard stop” tools, such as best practice advisory alerts that prevent clinicians from placing CT orders without confirming CDR use. Anticipating poor long-term acceptance of such alerts within our system, we instead adopted AgileMD, an optional-use embedded CDR software. Even without mandatory use, this intervention was temporally associated with reduced CT use across both sites, suggesting that accessible CDRs combined with clinician education can still promote evidence-based imaging reduction without the use of hard stop tools.

Because our academic department identified overuse of all-cause and low-risk trauma CT imaging as an opportunity for improvement, a modest investment was made to support the work. However, the reduction in CT orders would coincide with a reduction in unnecessary patient charges through this investment. Cost effectiveness would be an avenue for future research on similar QI campaigns.

The safety of reducing imaging in low-risk trauma was an essential consideration in this campaign. Review of the medical records for patients returning within 72 hours revealed no missed clinically important head or cervical spine injuries at either site, supporting the safety of our approach. This aligns with extensive evidence demonstrating that known head and cervical spine CDRs have high negative predictive values for clinically significant injuries and are safe to apply in routine practice. Our interventions including encouragement of known CDRs were also implemented within EDs that already maintain structured quality and safety oversight programs, including clinician-initiated case referrals, peer review processes, and regular morbidity and mortality conferences.

No adverse outcomes attributable to the campaign were identified through these mechanisms or chart review of patients returning within 72 hours who required admission. Although it is possible that some patients may have sought care at outside hospitals and, therefore, would not appear in our 72-hour return-admission review, we believe the likelihood of clinically important missed injuries escaping detection is low given that no such injuries were identified among patients who did return to our sites. Based on our experience, we recommend that EDs adopting similar QI efforts ensure that comparable safety review structures are in place to monitor unintended consequences.[Table t1-wjem-27-947]

Overall, our findings indicate that a multimodal intervention including clinician and patient education, consistent clinician feedback, and CDRs embedded in the EHR can effectively reduce head and cervical CT exams in low-risk trauma patients, while maintaining patient safety outcomes. Short-term results were promising, but ongoing data collection is needed to assess sustainability of our interventions. Some regression is expected with less-frequent feedback, yet several campaign elements remain in place. Patient education handouts and embedded CDRs continue to be used. Although the formal QI campaign ended to allow focus on other department priorities, these components are likely to sustain the positive trend in CT use for low-risk head and cervical spine trauma. The hope is that our educational elements and overall campaign have caused a long-term culture shift with respect to clinician use of CT in low-risk trauma patients.

## LIMITATIONS

Physicians practicing at the community site within this hospital system also work at and conduct research through the academic institution and may demonstrate ordering patterns similar to those at the academic site. Thus, it would be difficult to generalize our community ED findings to standalone community EDs. The study was not designed to examine clinicians’ practice patterns at the academic ED compared to the community ED but may represent an area of opportunity for future QI projects or research.

Additionally, there is no gold standard in the emergency medicine literature for the “correct” number of CT exams to order per 100 patients. Rates of CT use are governed by individual site-specific characteristics such as trauma center designation, individual patient characteristics (age, mechanism of injury, rate of anticoagulation, etc), and regional physician-ordering practices. Therefore, results from this study may not be generalizable to all EDs.

This study is also subject to bias, namely the Hawthorne effect. Clinicians may have changed their imaging practices simply due to the awareness of being observed, independent of the intervention’s impact. We sought to mitigate this bias by establishing a six-month pre-intervention baseline prior to introducing this project to clinicians and used the pre-intervention baseline to compare results of our post-intervention data with use of sensitivity analyses to ensure statistical significance. Moreover, because the study was not a randomized controlled trial, it was challenging to statistically assess the influence of individual interventions from this multimodal campaign. Future studies designed as randomized controlled trials or with methods to assess comparative effectiveness could provide more definite conclusions regarding the value and weight of individual intervention components.

## CONCLUSION

Using quality improvement science, a multimodal campaign integrating patient and clinician education, data feedback, and EHR decision support significantly reduced head CT and cervical spine CT use in low-risk trauma patients. These findings provide a translatable and scalable framework to address unnecessary imaging in the ED and promote evidence-based clinical decision-making in emergency care.

## Figures and Tables

**Table 1 t1-wjem-27-947:** Demographic characteristics of emergency department patients at the academic and community sites within our health system from January 2020–December 2024, including all patients who underwent head or cervical spine computed tomography imaging for low-risk trauma.

	All Patients		Low-Risk Trauma with HCT		Low-Risk Trauma with CSCT	
	Academic N_avg_ = 62900	Community N_avg_ = 42197	Academic N_avg_ = 2273	Community N_avg_ = 1993	Academic N_avg_ = 203	Community N_avg_ = 108
**Age in years**						
<18	619 (1.0)	844 (2.0)	12 (0.5)	14 (0.7)	2 (1.1)	2 (1.6)
18–40	27612 (43.9)	13432 (31.8)	756 (33.3)	235 (11.8)	84 (41.3)	33 (30.2)
41–64	23267 (37.0)	14787 (35.0)	743 (32.7)	348 (17.4)	78 (38.2)	49 (45.4)
>65	11371 (18.1)	13131 (31.1)	761 (33.5)	1396 (70.0)	39 (19.4)	25 (22.8)
Unknown	31 (0.05)	2 (0.01)	1 (0.03)	0 (0.0)	0 (0.0)	0 (0.0)
**Sex**						
Male	31897 (50.7)	18005 (42.7)	1311 (57.7)	793 (39.8)	103 (50.8)	42 (38.7)
Female	30990 (49.3)	24181 (57.3)	962 (42.3)	1199 (60.2)	100 (49.2)	66 (61.3)
Other	12 (0.02)	12 (0.03)	0 (0.0)	0 (0.0)	0 (0.0)	0 (0.0)
**Race**						
White	26551 (42.2)	28982 (68.7)	1313 (57.8)	1668 (83.7)	102 (50.2)	76 (70.8)
Black	29702 (47.2)	7212 (17.1)	781 (34.4)	162 (8.1)	83 (40.7)	18 (16.9)
Other	6036 (9.6)	5674 (13.4)	166 (7.3)	157 (7.9)	18 (8.8)	13 (12.1)
Unknown	611 (1.0)	329 (0.8)	13 (0.6)	6 (0.3)	1 (0.3)	1 (0.2)
**Ethnicity – Hispanic or Latino**	4121 (6.6)	2285 (5.4)	97 (4.3)	67 (3.4)	12 (6.1)	7 (6.2)
**Payor**						
Commercial	12394 (19.7)	13934 (33.0)	388 (17.1)	288 (14.5)	46 (22.8)	39 (35.8)
Medicaid	22018 (35.0)	7444 (17.6)	591 (26.0)	132 (6.6)	50 (24.5)	16 (14.4)
Medicare	16976 (27.0)	15008 (35.6)	824 (36.3)	1357 (68.1)	52 (25.7)	30 (27.9)
Self-pay	8575 (13.6)	4227 (10.0)	246 (10.8)	117 (5.9)	26 (12.7)	9 (8.2)
Other	2937 (4.7)	1584 (3.8)	223 (9.8)	99 (5.0)	29 (14.3)	15 (13.7)

Data presented as number (percentage).

*HCT*, head computed tomography; *CSCT*, cervical spine computed tomography.
